# Recent advances in host models in studying virulence factors and pathogenesis of *Acinetobacter baumannii*

**DOI:** 10.3389/fcimb.2025.1715193

**Published:** 2025-11-10

**Authors:** Tadesse Lejisa Garomsa, Abebe Mekuria Shenkutie, Polly H. M. Leung

**Affiliations:** 1 Department of Health Technology and Informatics, The Hong Kong Polytechnic University, Hong Kong, Hong Kong SAR, China; 2 Infectious Diseases Research Directorate, Ethiopian Public Health Institute, Addis Ababa, Ethiopia; 3 Department of Microbiology, Immunology, and Parasitology, St. Paul’s Hospital Millennium Medical College, Addis Ababa, Ethiopia

**Keywords:** *Acinetobacter baumannii*, virulence factors, animal models, cell culture, pathogenesis

## Abstract

*Acinetobacter baumannii* is a major global healthcare-associated pathogen with increased antibiotic resistance, underscoring the urgent need to develop novel antibiotics, immunotherapies, and vaccines. However, a critical gap in combating *A. baumannii* infections is the lack of a systematic approach to selecting appropriate host models for studying host-pathogen interactions. This review analyses previously published research articles on host interactions with *A. baumannii*. The articles were searched from electronic databases, including PubMed, ScienceDirect, and Google Scholar, covering publications from 2015 to 2024. These studies, encompassing both *in vitro* and *in vivo* research, aim to elucidate the virulence factors and host responses during host-pathogen interactions. The review analysis shows that mouse and *Galleria mellonella* are well-established animal models. Meanwhile, A549, HEK-293, HeLa, and Hep-G2 cell lines are the main *in vitro* models for studying *A. baumannii* pathogenesis. Key considerations for *in vitro* host model selection include necessitating a minimum of biosafety level 2 (BSL-2) laboratory conditions, the specific pathogens involved, relevance of the cell lines, immune responsiveness, and ease of manipulation. Challenges such as the heterogeneity of bacterial strains, initial bacterial dose, incubation duration, and infection multiplicity should be considered in host-pathogen studies. This review could serve as a roadmap for optimizing experimental models to accelerate the development of targeted strategies against *A. baumannii*.

## 1 Introduction

*Acinetobacter baumannii* is a healthcare-associated pathogen, Gram-negative, aerobic, catalase-positive, oxidase-negative, and nonfermenting coccobacilli (Yehya et al., 2025). It is a significant global health threat, which is an emerging pan-drug-resistant pathogen with high morbidity and mortality (Lim et al., 2019). It can survive for an extended time, particularly in hospital environments, on different surfaces such as beds, bedside tables, soft surfaces, and medical equipment (Alsan and Klompas, 2010). It also has the capacity to form resistant biofilms, which enables it to resist antibiotics and host immune responses (Sharma et al., 2023). These all increase the risk of transmission to patients and healthcare workers (Sharma et al., 2023). This bacterium is associated with healthcare-associated infections (HAIs)that primarily affect immunocompromised patients and can also lead to life-threatening conditions (Thacharodi et al., 2024). The most common diseases it causes are ventilator-associated pneumonia (VAP), followed by bacteremia, urinary tract infections (UTI), wound infections, skin infections, and meningitis (Abdallah and Abdalla, 2021).

It has been recently recognized that it is a challenging pathogen resistant to last-resort antibiotics, environmental stresses, hospital disinfectants, and host immune effector cells (Maure et al., 2023). To date, minimum antibiotic choices have demonstrated a significant ability to improve outcomes in patients with carbapenem-resistant *A. baumannii* (CRAB) infections (Bassetti et al., 2021; Yehya et al., 2025). Over the past decade, mortality rates for patients with CRAB infections are alarmingly elevated, ranging from 13.6% to 78%, mainly in patients with septic shock (Appaneal Haley et al., 2022; Boutzoukas and Doi, 2025; Russo et al., 2018). In 2021, the Global Burden of Disease (GBD) 2021 Antimicrobial Resistance Collaborators estimated that 1.14 million deaths were associated with deadly multidrug-resistant (MDR) bacteria, with *A. baumannii* being the most significant contributor (GBD 2021 Antimicrobial Resistance Collaborators, 2024). They forecasted that a total of 39.1 million people could die from antimicrobial-resistant (AMR) bacterial infections, including those caused by *A. baumannii*, between 2025 and 2050 (GBD 2021 Antimicrobial Resistance Collaborators, 2024). Thus, the WHO has ranked the CRAB as the top critical pathogen that requires new vaccines and drug developments (WHO, 2024).

To effectively combat this bacterium, it is crucial to understand the mechanisms that favor its survival under different conditions. According to previously published reports, the pathogen utilizes a range of virulence factors at various stages of pathogenicity, thereby favoring its survival under diverse conditions, including antibiotics, host immune responses, and environmental stresses (Karampatakis et al., 2024; Shadan et al., 2023). Several virulence factors of *A. baumannii*, including those involved in biofilm formation, drug resistance, cell adhesion, invasion, and persistence in the host, have been investigated (Lucidi et al., 2024). To uncover the pathogenesis of *A. baumannii*, scientists have extensively researched using various *in vitro* and *in vivo* experimental models. Various host models, including *in vitro* cell lines and *in vivo* animal models, have been employed to decipher the bacterial virulence mechanisms and host responses during *A. baumannii* infections. Despite the plethora of studies that leverage host models to investigate host-pathogen interactions, certain scientific questions, such as model relevance, remain unexplored in selecting appropriate host models. In addition, to date, there are no standardized laboratory methods for host-*A. baumannii* interaction studies. Accordingly, exploring different laboratory experiment-related factors that may contribute to the unsuccessful translation of preclinical studies to clinical studies is crucial. Thus, this review aims to provide an overview of the current progress of *in vitro* and *in vivo* host models used in studying host-pathogen interactions during *A. baumannii* infections. This review also highlights the challenges involved in study designs that may affect the translation of preclinical studies to clinical studies.

## 2 Methods

### 2.1 Search strategy and study selection criteria

This review article was conducted on published articles to summarize the progress in using host models to decipher virulence factors of *A. baumannii* and to emphasize the strain and methodological differences that may contribute to the unsuccessful translation of *A. baumannii* preclinical studies to clinical studies. A comprehensive literature search was conducted for this narrative review using the following electronic databases: PubMed, ScienceDirect, and Google Scholar. The search strategy included the following keywords in combination using Boolean operators (AND, OR): “*Acinetobacter baumannii,”* “*A. baumannii,”* “host models,” “host-microbe,” “host-bacterial,” “virulence factors,” “animal models,” and “cell lines” both in Medical Subject Heading (MeSH) and free text terms.

**Figure 1:**
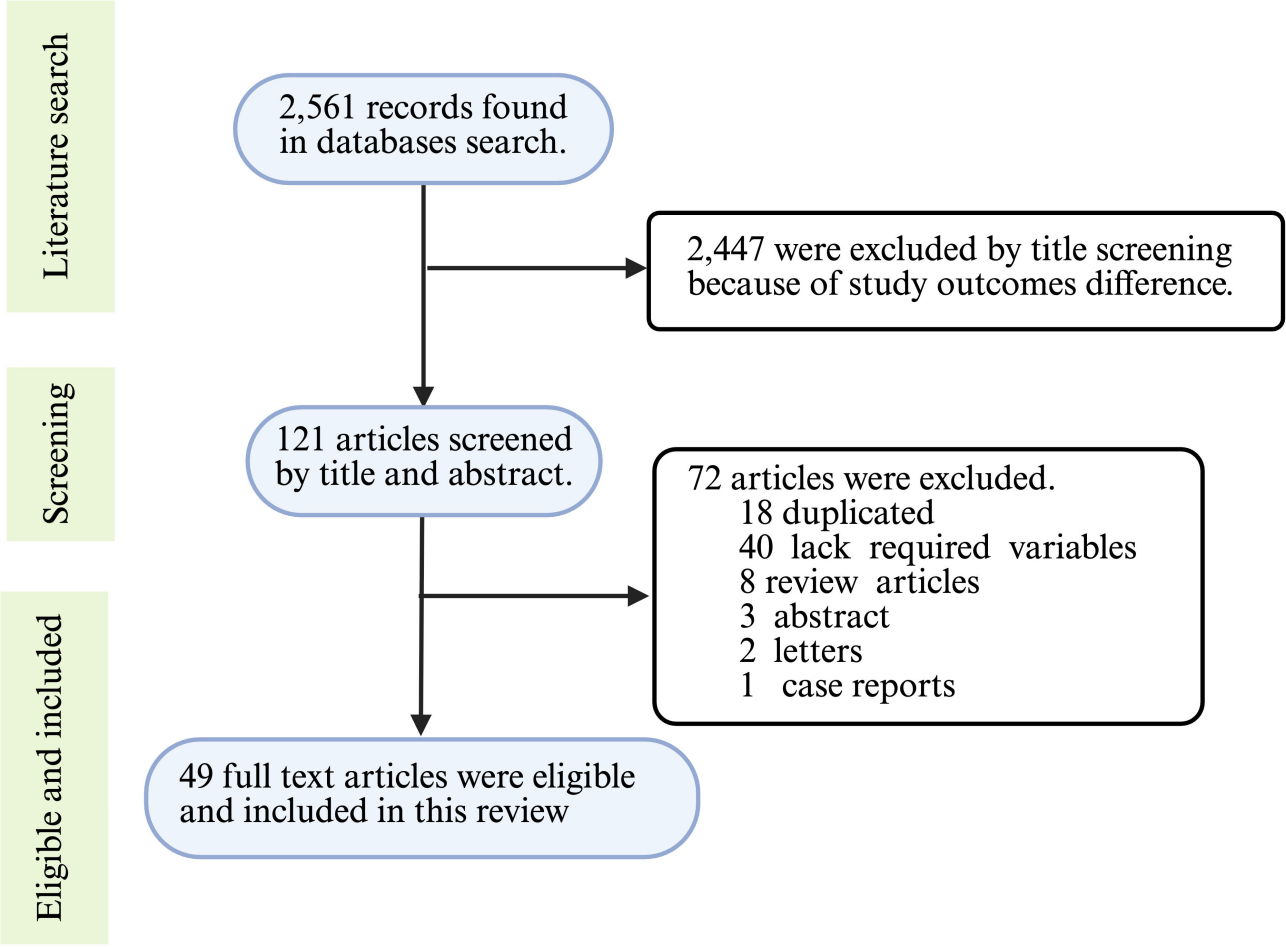
Article selection process flowchart. The data extracted from the selected articles included *in vivo* animal models, *in vitro* cell cultures, virulence factors of *A. baumannii*, the roles of virulence factors in pathogenesis, inoculum size, multiplicity of infection, and incubation and monitoring times. This information was synthesized and presented to provide a comprehensive overview of the current progress in host models for uncovering virulence factors of *A. baumannii*, with an emphasis on the challenges posed by strain and method heterogeneity. The figure was created with BioRender (https://www.biorender.com/).

The peer-reviewed original research articles published in English from 2015 to 2024 were included in this study. The articles relevant to the topic of host models used in studying *A. baumannii* virulence and pathogenesis were included. TLG and AMS carried out the article search and screening independently. Case report studies, conference abstracts, and duplicated articles were excluded from this study (**Figure 1**). During the initial search, the two reviewers independently identified 2,561 records through comprehensive searches of electronic databases. Numerous (2,447) articles were excluded by title screening. More articles were excluded due to duplications (18), case report (1), and conference abstracts (3) during the abstract screening process. Finally, 49 full articles were selected based on their relevance to this review article and the quality of the whole article (**Figure 1**).

## 3 Virulence factors and pathogenesis of *A. baumannii*

*A. baumannii* utilizes different arsenals of virulence factors, including outer membrane proteins (OMPs), lipopolysaccharide (LPS), lipooligosaccharide (LOS), pili, biofilm formation, capsular polysaccharides (CPS), metal ion acquisition systems, efflux pumps, two-component systems (TCSs), and secretion systems to favor its survival in the environment and host (Karampatakis et al., 2024; Shadan et al., 2023). In general, these virulence factors enable *A. baumannii* to adhere to and invade the host, evade the host’s immune response, proliferate, and successfully establish an infection in the host (Chen, 2020).

OMPs are beta barrel-shaped porins that facilitate the flow of tiny particles into and out of the periplasmic space of Gram-negative bacteria, including *A. baumannii* (Slusky and Dunbrack, 2013). They are major immunogenic proteins in *A. baumannii*, helping in bacterial environmental stress tolerance, antibiotic resistance, and host immune evasion. Immunization with *A. baumannii* OMPs resulted in a significant increase in protective immune responses (Mirali et al., 2023). Passive antibodies against OMPs of *A. baumannii* also safeguarded experimental animals from disease (Rafiei Delfan et al., 2024; Rajabzadeh et al., 2024). OmpA, CarO, outer membrane carboxylate channels, OmpW, and Omp33–36 are the major outer membrane proteins involved in different pathogenesis roles. Lipopolysaccharide and lipooligosaccharide, the *A. baumannii* surface adhesins, are important virulence factors (Eijkelkamp et al., 2014). Various genes of LPS and LOS of *A. baumannii* have been reported to activate Toll-Like Receptors (TLRs) and trigger the host innate immune response (Moffatt et al., 2013).

Capsular polysaccharide is a critical virulence factor responsible for resisting complement-mediated killing and evading phagocytosis by host immune cells (Zierke et al., 2025). More than 100 types of capsule loci have been identified from *A. baumannii* (Shashkov et al., 2017) which substantially influences virulence. Pili are found on the surface of both Gram-negative and Gram-positive bacteria. In *A. baumannii*, they play a crucial in facilitating attachment, biofilm formation, and twitching and surface-associated motility, employing different genes involved in various pathogenesis roles (Ahmad et al., 2023). Most *A. baumannii* strains express genes involved in biofilm formation and associated with the virulence level of the bacterium (Wiradiputra et al., 2025). Mostly, these genes include biofilm-associated protein (BAP) and pili genes (Wiradiputra et al., 2025). The efflux systems and transcriptional regulators have been reported to regulate biofilm formation (Vareschi et al., 2025). These genes are involved in biofilm growth, and the biofilm, in turn, helps with adhesion and drug resistance (Vareschi et al., 2025).

Efflux pumps are also important *A. baumannii* virulence factors involved in virulence, extruding the antibiotics from bacterial cells and surface-associated motility (Pérez-Varela et al., 2019). The efflux pump has six classes with different roles in pathogenesis. The major facilitator superfamily (MFS) and resistance nodulation division (RND) are mainly involved in the resistance of multiple classes of antibiotics (Fournier et al., 2006). *A. baumannii* can uptake essential metal ions from the host for its survival using different metal ion acquisition mechanisms. Iron (Fe²^+^) and manganese (Mn²^+^) ions are the most critical and essential metal ions for the survival of *A. baumannii*. This bacterium uses siderophores to uptake Fe²^+^ (Gaddy et al., 2012) and MumT (manganese and urea metabolism transporter) to uptake Mn²^+^ (Green et al., 2020). This system can be used to help in introducing specific antibiotics inside bacteria by using the Trojan Horse strategy using specific siderophores to fight antimicrobial resistance in pathogenic bacteria, including *A. baumannii* (Tillotson, 2016). Thus, targeting the metal acquisition systems can be a future perspective to fight antimicrobial resistance in pathogenic bacteria, including *A. baumannii* (Ezzeddine and Ghssein, 2023). Another important virulence factor discovered in *A. baumannii* is the secretion systems. So far, five secretion systems, namely type I secretion system (T1SS), type II secretion system (T2SS), type IV secretion system (T4SS), type V secretion system (T5SS), and type VI secretion system (T6SS), have been identified. The T5SS and T6SS play a crucial role in *A. baumannii* pathogenesis, including biofilm formation, attachment to host cells, delivery of toxic effector proteins to host cells or neighboring bacterial cells, and survival in systemic infection.

**Figure 2:**
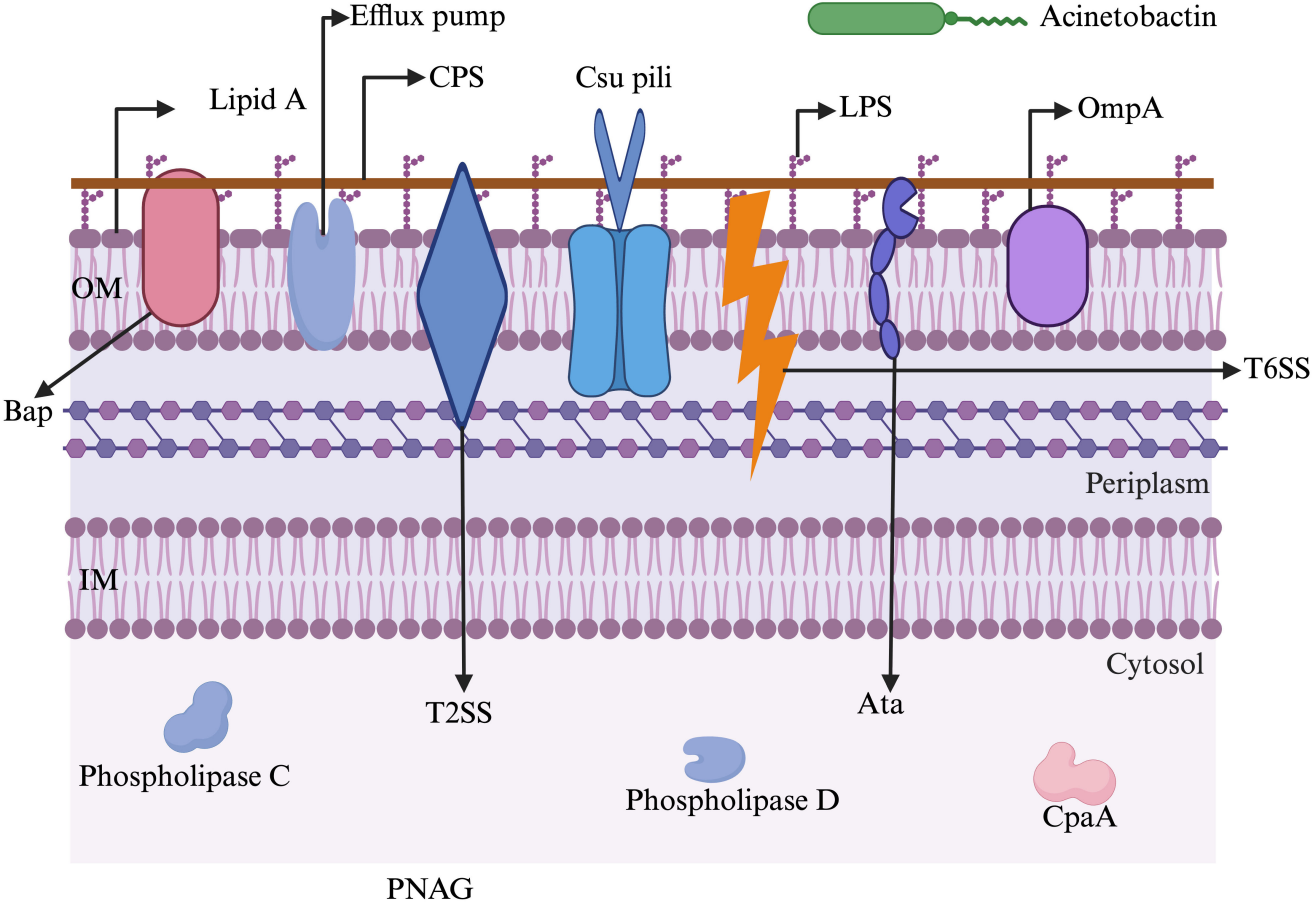
A schematic representation of major virulence factors possessed by *A. baumannii* to withstand harsh environments and establish infection. Acinetobactin: involved in nutrition/metal uptake; Ata: *Acinetobacter* trimeric autotransporter (involved in adherence to host membranes); Bap: biofilm-associated protein (involved in maturation of biofilms); CpaA: Coagulation targeting metallo-endopeptidase of *A. baumannii* (involved in blood coagulation); CPS: capsular polysaccharide (involved in immune modulation, antiphagocytosis); Csu pili: chaperon/usher pilus system (involved in biofilm formation and adherence); Efflux pump: involved in antibiotic resistance and biofilm formation; IM: inner membrane; LPS: lipopolysaccharide (involved in evasion of the host immune response and triggering the host inflammatory response); OM: outer membrane; OmpA: outer membrane protein A (involved in apoptosis, biofilm formation, avoiding complement-mediated killing, and adherence and invasion of epithelial cells); Phospholipase C: involved in lysis of host cells; Phospholipase D: aids in bacterial persistence in human serum; PNAG: poly-β-1,6-N-acetylglucosamine (aids in biofilm formation and adhesion); T2SS: type II secretion system (aids in secreting multiple effectors); T6SS: type VI secretion system (helps in delivering protein effectors and bacterial competition). The figure was created with BioRender (https://www.biorender.com/).

In general, multiple genes within specific arsenals of virulence factors are involved in the pathogenesis process of *A. baumannii*. The pathogenicity roles of some crucial genes are illustrated in **Tables 1**-**3**. **Figure 2** illustrates the key virulence determinants of *A. baumannii* that play various roles in pathogenesis.

**Table 1:** Virulence factors and pathogenicity of *A. baumannii* in host cell line models.

Virulence factor	Important findings in pathogenesis	Cell line	Multiplicity of infection	References
OmpA	The addition of anti-*OmpA* substantially reduced the adherence and invasion ability of *A. baumannii* strains and improved cell viability.	HeLa	100	(Rafiei Delfan et al., 2024)
Omp34	In the presence of anti-Omp34, bacterial adherence and internalization have been attenuated, and the viability of A549 cells was improved.	A549	100	(Rajabzadeh et al., 2024)
*CarO*	The strain with Δ*carO* mutants revealed a reduced ability to adhere to and invade A549 cells.	A549	1000	(Labrador-Herrera et al., 2020)
*OmpW*	The loss of *OmpW* significantly affected the adherence and invasion of *A. baumannii* in A549 cells compared to the WT strain at 2 and 24 hpi.	A549	500	(Gil-Marqués et al., 2022)
SurA1	20 μg/mL of SurA1 exhibited 10% of A549 cell death & 15% of HEp-2 cell death. 40 and 80 μg/mL of SurA1 showed high cytotoxic effects on both cells.	HEp-2 and A549	0, 20, 40, or 80 μg/mL of rSurA1 in 10^4^ cells	(Liu et al., 2016)
*A. baumannii* genes	Deletion of *feoA*, *basB*, *esvF2*, and *bfnL* exhibited a significant reduction in the ability of bacterial strains to adhere to A549 cells.	A549	100	(Martinez-Guitian et al., 2021)
*clp*	The knocking out of the *clpA*, *clpB*, and *clpS* genes reduced *A. baumannii* survival by 41.2%, 62.3%, and 73.2% compared to the WT.	J774	10	(Belisario et al., 2021)
*TrmB*	The Δ*trmB* mutant exhibited a replication defect in macrophages, which persisted within the cells for only 4 hours.	J774	10	(McGuffey et al., 2023)
*gigAB*	The BMDMs infected with *ΔgigAB* exhibited a 300-fold reduction in live bacteria in BMDMs.	Mouse BMDM	10	(Zhou et al., 2021)
*Ata*	*A. baumannii* producing *Ata* adhesin significantly adhered to different host cells compared to the *Ata*-deficient strains.	HUVEC, A549, HeLa, HepG-2	200	(Weidensdorfer et al., 2019)
*plcN and lasB*	The presence of any of these genes increased the invasiveness and killing capability of the *A. baumannii* strain when infecting the A549 cell line.	A549	200	(Kareem et al., 2017)
*tagP*	Significantly lower adherence was exhibited in *ΔtagP* mutant strains when compared to the WT strain	HBEpiC and A549	20	(Li Y. et al., 2024)
*abaI*	The *ΔabaI* mutant strain demonstrated a lower ability to adhere and invade the host cells, which was regained with the complemented strain.	A549	100	(Jiang et al., 2024)
trxA	ΔtrxA OMVs were more cytotoxic to cells than WT OMVs, which was diminished when OMVs were treated with protease K, indicating that the cytotoxicity was related to other OMV proteins.	J774 cells	1 μg or 10 μg OMV with 2.5 x 10^5^ cells	(Shrihari et al., 2022)
*InvL*	The Δ*invL* mutant strain demonstrated a significantly lower capacity to adhere to both kidney and bladder cells than the WT strain.	MDCK and 5637	1	(Jackson-Litteken et al., 2022)
*sRNA 13573*	The WT AB17978 strain significantly attached to A549 cells compared to the *ΔsRNA13573* strain.	A549	Not reported	(Alvarez-Fraga et al., 2017)
*A1S_0114*	The parental 17978 adhered significantly to epithelial cells than the *Δ0114* mutant strains.	A549	10	(Rumbo-Feal et al., 2017)
*blp1*	The *blp1* deletion resulted in a substantial reduction of adhesion of the *AbIC IΔblp1* strain to epithelial cells by threefold.	LL/2	1000	(Skerniškytė et al., 2019a)
*wza*	The *Δwza* strain exhibited a drastic increase in phagocytosis by macrophages.	J774	200	(Skerniškytė et al., 2019b)
*wza*	The *wza* knockout strains showed substantially lower adhesion to A549 cells compared to the WT strains.	A549	Not reported	(Niu et al., 2020)
*FhaBC*	Deletion of *the FhaBC* gene significantly reduced the adherence of the *AbH12O-A2* strain to host cells.	A549	10	(Pérez et al., 2017)

BMDM, bone marrow-derived macrophage; OMV, outer membrane vesicles; WT, wild type.

**Table 2:** Virulence factors and pathogenicity of *A. baumannii* in *G. mellonella* animal model.

Virulence factor	Important findings in pathogenesis	Injected inoculum size* (CFU/larva)	Monitoring time* (Days)	Reference
*OmpR*	A *ΔompR* mutant strain reduced the mortality of larvae by 40% at 72 hours.	1 x 10^5^	3	(Trebosc et al., 2022)
*lpxB*	The synergy effect of anti*-lpxB* pPNA with colistin increased the survival rate of *G. mellonella* compared to larvae treated with colistin or anti-*lpxB* pPNA only.	3 x 10^3^	4	(Martinez-Guitian et al., 2020)
*Ata*	Injection of the Δ*ata* mutant strain improved the survival rate of larvae by 30% after 24 hrs, whereas 100% died by the WT strain.	3 x 10^7^	1	(Weidensdorfer et al., 2019)
*tssD*	The larvae infected by the *ΔtssD* mutant strain revealed a significantly higher survival rate on the 4^th^ day of infection.	1 x 10^5^	4	(Li P. et al., 2024)
*tagP*	The survival rate of *G. mellonella* infected with the WT strain was 45%, while it was more than 80% when infected with the Δ*tagP* mutant strains.	3 x 10^6^	4	(Li Y. et al., 2024)
*adeAB*	100% of the larvae infected with the WT strain S1 died at 24 hpi, while more than 50% survived when infected with the *ΔadeAB* mutant strain at 120 hpi.	1 x 10^6^	5	(Richmond et al., 2016)
*SurA1*	>90% of WT strain injected larvae died, whereas <30% of the larvae infected with the *ΔSurA1* mutant strain died at 2 days of infection.	1 x 10^4^	5	(Liu et al., 2016)
*abaM*	The virulence of the *ΔabaM* mutant strain was totally diminished in a *G. mellonella* infection model.	2 x 10^4^	5	(López-Martín et al., 2021)
*Tol-Pal* genes	The deletion of the *tolQ*, *tolR*, *tolA*, *tolB*, and *pal* genes from the ATCC 19606 strain exhibited reduced virulence in the *G. mellonella* infection model.	1 x 10^6^	7	(Hubloher et al., 2023)
*gigAB*	Rapid killing of *G. mellonella* was observed at 8 hpi when infected with wild-type 17978, whereas no larvae died at 48 hpi when infected with the *ΔgigAB* strain.	5 x 10^6^	2	(Zhou et al., 2021)
*plcN* and *lasB*	Harboring the *plcN* and *lasB* genes enhanced the killing capacity of *A. baumannii* strains.	1.6 x 10^6^	8	(Kareem et al., 2017)
Capsule *(KL type)*	The KL120 bacterial-infected larvae caused the quickest and most unforeseen death compared to those infected with KL2, KL7, and KL9.	1 x 10^6^	3	(Domingues et al., 2024)
*Oxa-822*	About 91% of meropenem-treated larvae survived when infected with the *OXA-822*-deficient strain, while only 52.7% survived when infected with the *OXA-822*-harboring strain.	5 x 10^5^	3	(Tietgen et al., 2021)
*A1S_0114*	The virulence of the *Δ0114* mutant strain was substantially attenuated in *G. mellonella* compared to the WT.	1 x 10^5^	5	(Rumbo-Feal et al., 2017)
*lpxO*	Only 10% of the larvae survived when infected with the WT and complemented strains, whereas 50% of the larvae survived when challenged with the *ΔlpxO* strain.	5 x 10^4^	3	(Bartholomew et al., 2019)
*wza*	Mortality of *G. mellonella* reached 73.3% when challenged with the WT strain, whereas it was 26.7% when infected with the *Δwza* strain on 2 days post-infection.	1 x 10^6^	7	(Niu et al., 2020)
Iron acquisition system	The deletion of iron acquisition genes *bfmS, gigD*, and *zur* significantly attenuated the virulence of the AB5075 strain in *G. mellonella.*	1 x 10^6^	6	(Gebhardt et al., 2015)

* Different inoculum sizes were used at various monitoring times to investigate the functions of different virulence factors (indicated in **Table 2**). The virulence factors showed various degrees of killing, ranging from 10% to 100%. CFU, colony-forming unit; WT, wild-type.

**Table 3:** Virulence factors and pathogenicity of *A. baumannii* in the mouse animal model.

Virulence factor	Important findings in pathogenesis	Injected inoculum size (CFU/mouse)	Evaluation days (Days)	Reference
*CarO*	A 40% survival rate in mice infected with the *ΔcarO* mutant 17978 significantly lowered the bacterial burden in all organs.	1 x 10^3.2^	7	(Labrador-Herrera et al., 2020)
OmpA	A higher antibody titer was induced in OmpA-injected mice. OmpA administration in mice caused none to mild histopathological effects in different tissues.	100 μl of 100 μg rOmpA	30	(Rafiei Delfan et al., 2024)
Omp34 and BauA	There were 100% survival rates in mice injected with Omp34 and BauA. A substantial reduction in the bacterial load in the organs of vaccinated mice was observed.	6.7 x 10^7^	4	(Mirali et al., 2023)
*DksA*	A Δ*dksA* mutant strain showed less bacterial burden and mild histopathological changes in the lungs of mice.	5 x 10^8^	5	(Kim et al., 2021)
*TrxA*	A Δ*trxA* mutant Ci79 strain showed a significant reduction of bacterial burdens in the lungs, liver, and spleen. All mice challenged with Δ*trxA* survived.	1 x 10^8^	1	(May et al., 2019)
*trmB*	The virulence of Δ*trmB* was attenuated in a mouse model. Significant reduction of bacterial load in the tested organs.	5 x 10^7^	1	(McGuffey et al., 2023)
*Csu pili*	Little to no colonization of *ΔcsuA and ΔcsuA/B* mutants AB17978 in the lungs, liver, and spleen of mice was observed.	1 x 10^7^	1	(Ahmad et al., 2023)
*Pal*	The mice infected with the Δ*pal* mutant strain showed decreased mortality, with 80% of mice surviving. 40% of recombinant (rPal) immunized mice survived when infected with the WT strain.	5 x 10^6^	7	(Zeng et al., 2023)
Acinetobactin genes	Mice infected with Δ*entA, ΔbasG, ΔbasG, ΔbasD*, and *ΔbasB* mutant strains exhibited 90% survival rates.	7.5 x 10^7^	5	(Conde-Perez et al., 2021)
*Clp*	The mice infected with the Δ*clpA*, Δ*clpB*, and Δ*clpS* mutant strains showed 100%, 60%, and 80% survival rates at 35 days post-infection, respectively. The bacterial load in the lungs and kidneys of the mice also decreased.	5 x 10^6^	35	(Belisario et al., 2021)
*A1S_0114*	The bacterial loads in the lungs of mice infected with *Δ0114* were significantly lower compared to those of mice infected with 17978.	5.5 x 10^7^	2	(Rumbo-Feal et al., 2017)
*blp1*	The bacterial loads in the spleen of mice infected with the *Δblp1* strain showed a threefold reduction compared with those infected with the parental strain.	9.5 x 10^5^	7	(Skerniškytė et al., 2019a)
*bfmS*	About 60% of the mice infected with the *ΔbfmS* strain died at a dose that was completely survived with the WT strain.	7.5 x 10^7^	3	(Geisinger et al., 2018)
*gigC*	About 80% of mice infected intranasally with the *ΔgigC* mutant strain survived for 6 days, whereas 100% of those challenged with the WT strain died by the third day post-infection.	1 x 10^6^	6	(Gebhardt et al., 2020)
*wza*	Approximately 70% of mice infected with the WT strain succumbed, whereas only 20% died when challenged with the *Δwza* strain two days post-infection.	1 x 10^8^	7	(Niu et al., 2020)
*HisF*	The survival rate was markedly higher in mice infected with the *ΔhisF* strain compared to those infected with the parental strain.	7.5 x 10^8^	7	(Martínez-Guitián et al., 2019)
*FhaBC*	The mortality of mice infected with *the ΔFhaBC* strain was significantly reduced compared to when infected with the WT strain.	4.6 x 10^8^	7	(Pérez et al., 2017)
*mumT*	The *ΔmumT* burdens in the lungs and livers of mice were substantially lower than WT burdens.	5 x 10^8^	1.5	(Juttukonda et al., 2016)

The mice used in the above-mentioned studies were challenged with different *A. baumannii* strains. Different colony-forming units (CFU) of bacterial strains were used to challenge the mice and monitored for different hours or days. WT, wild-type.

## 4 Host models in studying *A. baumannii* virulence

Since *A. baumannii* is the top priority human pathogen requiring urgent new drug development, studying the pathogenic mechanisms involved during the infection process is crucial for identifying potential therapeutic targets. Various host models, including *in vitro* cell lines and *in vivo* animal models, have been reported for studying host and pathogen interactions.

### 4.1 *In vitro* cell culture models

Compared to animal models, cell culture is more convenient because it is easier to manipulate. The primary use of *in vitro* cell culture models is to create model systems for studying basic cell biology, reproducing disease mechanisms to mimic *in vivo* activity, or examining the toxicity of new drugs, vaccines, or immunotherapies (Segeritz and Vallier, 2017). Investigating host-pathogen interactions is crucial for understanding host cellular responses to infections. Nowadays, various cell cultures have been established to study a wide range of assays related to host-pathogen interactions, including 2D and 3D cell cultures (Park et al., 2021). The 2D cell culture models include immortalized cell lines that grow as a monolayer on flat surfaces, such as Petri dishes and flasks. In contrast, 3D cell cultures grow in three-dimensional structures, which have limited growth and higher costs. To date, different types of cell models, such as 2D-immortalized cell lines, primary cells, 3D-organoids, 3D-spheroids, and organs-on-a-chip, have been developed for infectious disease studies.

#### 4.1.1 Cell line models

**Figure 3:**
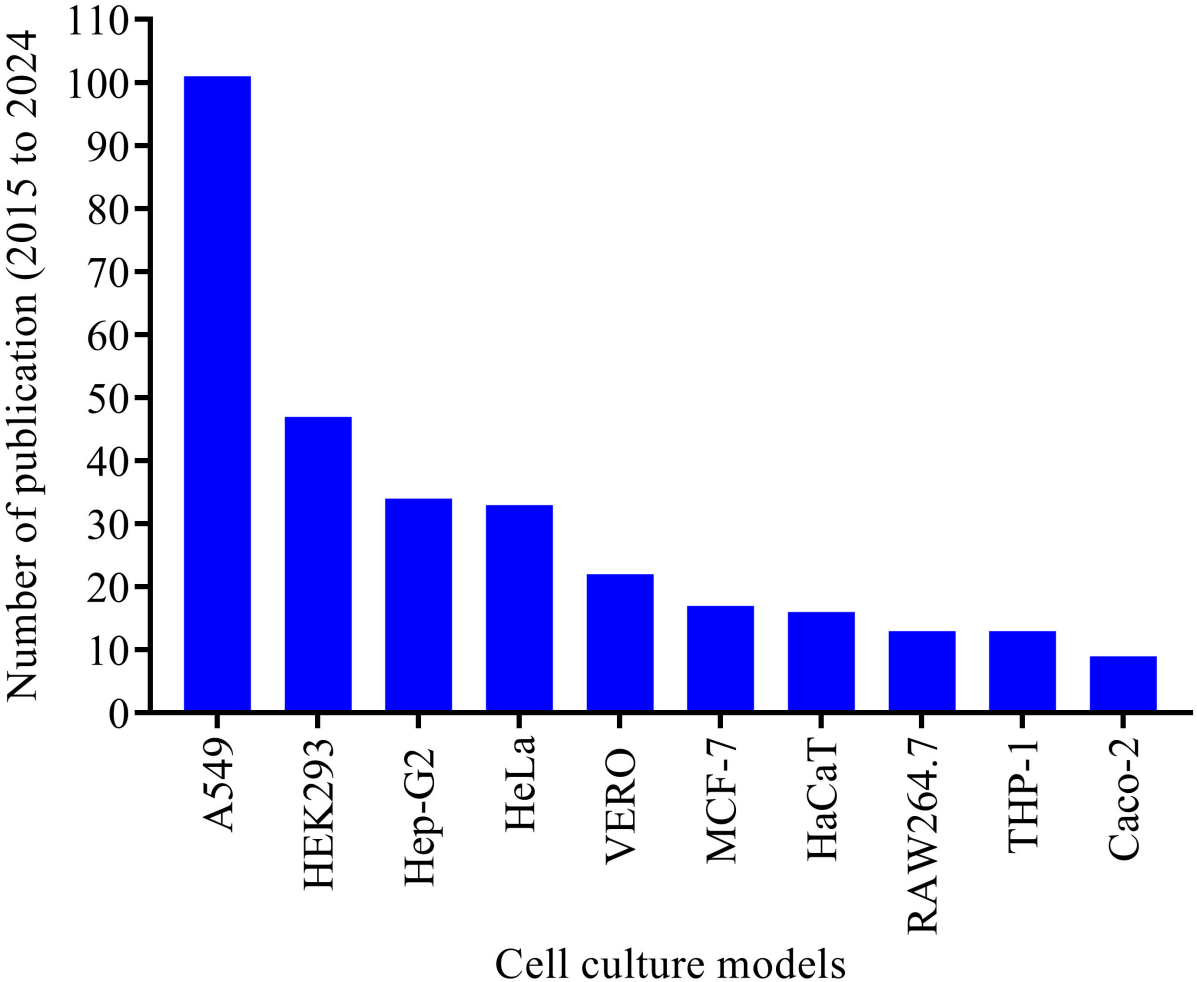
Top ten cell culture models tested and established in studying *A. baumannii* pathogenesis over the last decade (2015 to 2024). The electronic database search was made for the period from 2015 to 2024. More than 60 cell culture models were extracted, and only the top ten cell models extensively used in the last decade were illustrated in this figure.

*In vitro* cell lines are easier to be manipulated, they divide continuously and can be cultured for long periods of time. Cell lines can either be finite or continuous immortalized cells. Most of the cell lines that have been implemented in investigating host *A. baumannii* interactions so far are continuous immortalized cells (**Figure 3**). Over 60 cell culture models have been used in studying *A. baumannii* pathogenesis, including adhesion and invasion, cellular viability and toxicity, and cellular immune responses to the bacteria. The human alveolar basal epithelial cell (A549) was the most successful and extensively used immortalized cell line in the last decade, with more than 100 publications. Other commonly used cell lines include the human embryonic kidney (HEK-293) cell, human cervical carcinoma (HeLa) cell, and hepatocellular carcinoma (HepG2) cells were also commonly used cell lines in studying *A. baumannii* pathogenesis (**Figure 3**). The immune system-derived cells are crucial in studying infection-related inflammatory responses. The RAW 264.7 macrophage cell line, HL-60, and THP-1 monocytic cells were among the most used in modeling disease-associated inflammatory pathways (Barron et al., 2021). Over the last decade, because they are well characterized and stable, RAW 264.7 macrophage and THP-1 monocytic cells have gained significant attention from scientists and have been extensively utilized in studying inflammatory pathways in response to *A. baumannii* infection. The top ten established cell lines used in the last decade to study *A. baumannii* pathogenesis are summarized in **Figure 3**.

During the study of *A. baumannii* pathogenesis, cell culture models were widely used to investigate various virulence factors of *A. baumannii*. Bacterial adherence and internalization, host cell viability or cytotoxicity, and host cell immune response are among the most assessed parameters in cell culture models [**Table 1**]. For instance, *OmpA* has been evaluated for its effectiveness as a vaccine candidate, and *A. baumannii* adherence, internalization, proliferation, and viability assay were assessed on the HeLa cell line in the presence and absence of anti-OmpA serum, highlighting that *OmpA* plays a crucial role in *A. baumannii* epithelial cell interactions (Rafiei Delfan et al., 2024). The *A. baumannii* strain with the mutant *CarO* showed a reduced ability to adhere to and invade A549 cells, indicating that *CarO* is an important virulence factor and a potential target for new drugs against CRAB (Labrador-Herrera et al., 2020). Furthermore, the deletion of chaperone protease-encoding genes, such as *clpA, clpB*, and *clpS*, affected the survival and proliferation of *A. baumannii* in J774.16 cells (Belisario et al., 2021), indicating that these genes are essential for bacterial proliferation within host cells.

Additionally, QS genes are essential in aiding the adherence and invasiveness of *A. baumannii*. According to the report by (Tang et al., 2020), strains carrying *abaI* and *abaR* genes or producing acyl-homoserine lactone (AHL) signaling molecules exhibited substantial levels of adherence and invasion into A549 cells compared to strains lacking the QS genes or AHL signaling molecules. Furthermore, the *Acinetobacter* trimeric autotransporter adhesin, *Ata*, is reported to be an essential virulence factor involved in the adhesion of *A. baumannii* to host cells, as *Ata*-producing strains adhered more efficiently to the epithelial and endothelial cells compared to *Ata*-deficient strains (Weidensdorfer et al., 2019). Some recombinant proteins were also assessed for cytotoxicity on different cell lines (**Table 1**). For instance, rSurA1 was exposed to A549 and Hep-2 cells at varying concentrations, including 20, 40, and 80 μg/mL. The results showed that, 20 μg/mL caused 10% cell death in A549 and 15% in Hep-2 cells, whereas 40 and 80 μg/mL of rSurA1 were highly cytotoxic to both cells (Liu et al., 2016), indicating that SurA1 is a critical virulence factor in *A. baumannii* infection.

The 2D immortalized cell lines are inexpensive, easy to handle, and enable high-throughput screening of drugs and virulence factors. Despite this, because most cell lines are immortalized, they are unable to fully replicate the *in vivo* cell conditions, particularly those involved in host-pathogen interactions (Barron et al., 2021). Thus, primary human immune cells are crucial for studying host-pathogen interactions, as they closely mimic human body physiology.

#### 4.1.2 Primary cell models

Primary human immune cells have been developed and are used to address challenges in cell lines when studying the interaction between *A. baumannii* and host immune cells. These include primary monocytes, alveolar and interstitial macrophages, bone marrow-derived macrophages (BMDMs), dendritic cells, and neutrophils (Lazaro-Diez et al., 2020; Sabatini et al., 2024; Wang H. et al., 2024). When these cells are exposed to *A. baumannii*, they produce cytokines and/or chemokines. For instance, neutrophils infected with *A. baumannii* produce proinflammatory cytokines, such as IL-6, IL-1β, and CXCL-8, in response to this bacterium (Lazaro-Diez et al., 2020). Macrophages and monocytes have been shown to mediate bacterial clearance, while dendritic cells produce cytokines and chemokines (Sabatini et al., 2024). Alveolar and interstitial macrophages can undergo polarization toward the M1 phenotype, which can trigger a cytokine storm and usually cause the death of the host (Wang H. et al., 2024). This cytokine storm could be due to the high virulence level of the strain, which hyperactivates the host immune cells to produce high levels of proinflammatory cytokines and chemokines.

Virulence factors of *A. baumannii* are involved in evading the host’s immune responses, enabling them to survive and proliferate within immune cells. For example, a 10-fold reduction in intracellular live bacteria occurred at 2 hours post-infection (hpi) when BMDMs were infected with the wild-type (WT) 17978 strain. In contrast, a 300-fold reduction was observed at 2 hpi when infecting BMDMs with the *ΔgigAB* mutant strain. To verify this observation, the complementation strain displayed survival and replication levels comparable to those of the WT strain (Zhou et al., 2021). **Table 1** represents the key virulence factors examined in cell lines and primary cells over the past decade. It illustrates the virulence factors elucidated and their roles in pathogenesis across different cell lines. Different multiplicities of infection (MOI) and cell lines have been employed to assess the pathogenesis roles of the virulence factors. Various concentrations of purified proteins, but not the bacterial strains, were used to infect the cell lines to evaluate the cytotoxic effects of SurA1 and trxA.

#### 4.1.3 3D organoids, spheroids, and organs-on-a-chip models

To address the limitations of 2D cell culture models, such as their lack of complexity and physiological relevance, the 3D cell culture model has gained attention from the scientists in the recent years. The 3D cell cultures of organoids, spheroids, and organs-on-a-chip are emerging as advanced *in vitro* cell culture technologies for elucidating the pathophysiology of infectious diseases. Organoids are 3D cell cultures formed from induced pluripotent stem cells (iPSCs), embryonic stem cells (ESCs), or adult stem cells (ASCs) (Huch and Koo, 2015). They form cellular organization through self-renewal. So far, numerous organoids have been generated from human and mouse origins. They are genetically stable, can be manipulated indefinitely, and can be frozen for future use. To date, a few organoids, such as lung organoids for respiratory bacterial and viral infections, gastric and intestinal organoids for gastrointestinal infections caused by bacteria and viruses, and cerebral organoids for brain infections caused by viruses, have been well established. Other organoids for elucidating infectious diseases are in a very early stage.

Spheroids are spherical self-assembling 3D cell culture models, or are forced to grow as cell aggregates from single-cell suspensions (Yamada and Cukierman, 2007). These models are primarily used in cancer research, drug discovery, and regenerative medicine. They are rarely applied in infectious disease research. Even though lung spheroids are used to elucidate SARS-CoV-2 pathogenesis, they have not been harmonized for bacterial infection studies (Medina et al., 2025; Rosa et al., 2021). Organs-on-a-chip are emerging technologies that integrate organoids and organ chips to simulate relevant pathophysiology. Respiratory-on-a-chip, gut-on-a-chip, vasculature-on-a-chip, liver-on-a-chip, and kidney-on-a-chip are organ-on-a-chips utilized by infectious disease research so far. They have been employed to elucidate the pathophysiology of a very limited number of bacteria and viruses. None of these 3D organoids, spheroids, and organs-on-a-chip models has been established to study the pathogenesis of *A. baumannii*.

While 3D cell models have advantages over 2D cell models, they have some limitations, such as a lack of vascular and immune systems, low reproducibility, a lack of newly developed biomaterials (Williams, 2022), complexity of biofabrication technologies (Skardal et al., 2015), expense, and difficulty in setting up. In a natural disease state, various cell types can simultaneously interact with the pathogen to clear it from the host, whereas *in vitro* cell culture models typically utilize only a limited number of cell types, which cannot fully mimic the host’s immune response. Thus, as *in vitro* cell models are the first step in deciphering the virulence factors involved in host-pathogen interactions, it is crucial to validate the *in vitro* results with *in vivo* host models to examine hypotheses that might be translated into clinical settings.

### 4.2 *In vivo* nonmammalian animal models

**Figure 4:**
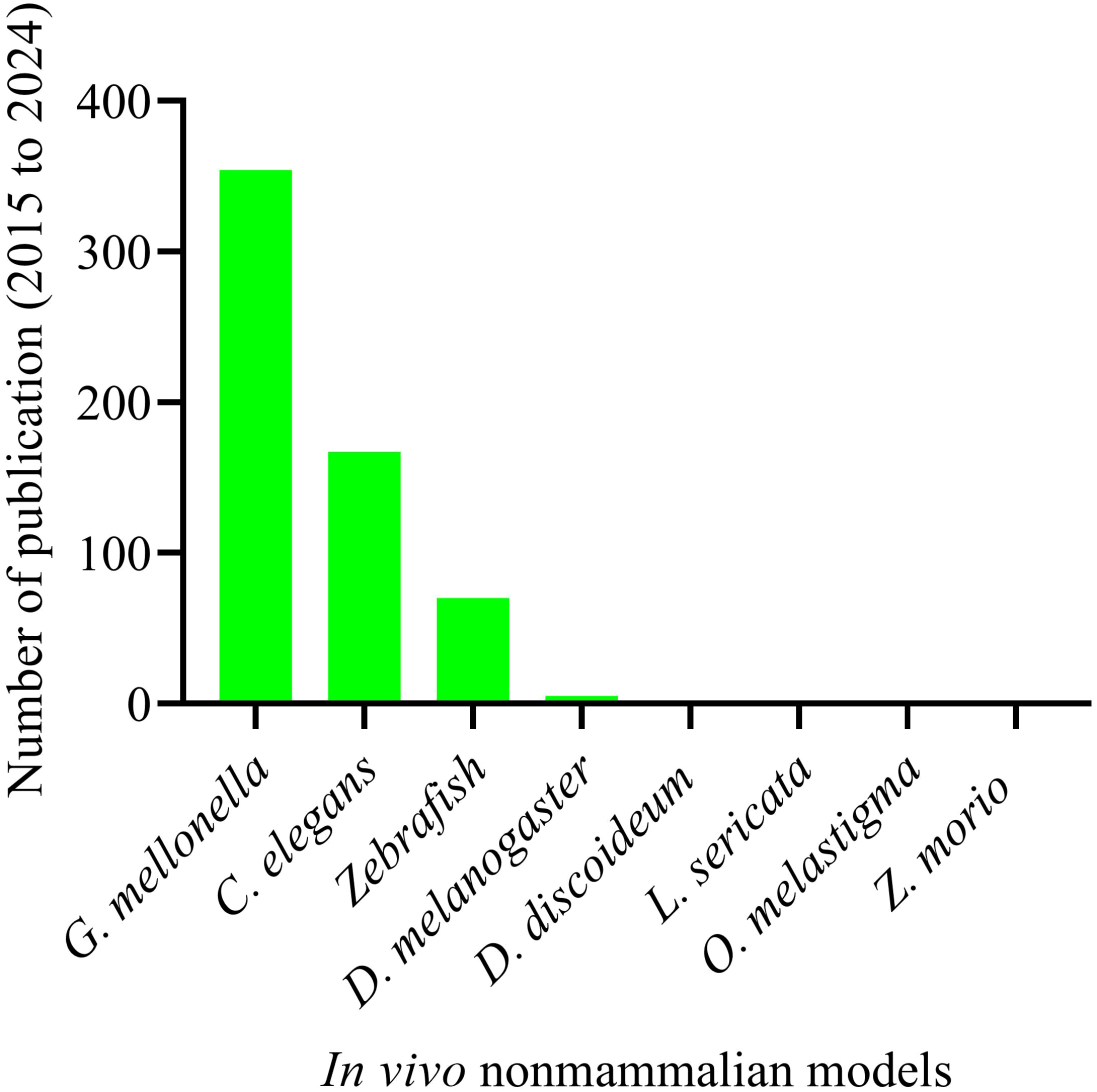
Non-mammalian animal models tested and established in studying *A. baumannii* pathogenesis over the last ten years. Total number of publications on non-mammalian animal host models and *A. baumannii* pathogenesis. Animal models: “*G. mellonella*” (354); “*C. elegans*” (167); “Zebrafish” (70); “Drosophila” (5); “*D. discoideum*” (2); “*L. sericata*” (2); “*O. melastigma*” (1); and “*Z. morio*” (1). Systematic search was made for the period from 2015 to 2024.

The complex and dynamic nature of host-microbe interactions presents significant challenges for fully replicating and elucidating these processes within *in vitro* cell culture models (Lemaitre and Ausubel, 2008). To address these challenges, numerous non-mammalian animal models have been tested and established to study *A. baumannii* pathogenesis. These models are crucial to the proof of concept for *in vivo* activity, enabling scientists to identify the key virulence factors responsible for host damage. To date, *Galleria mellonella* is the most widely used non-mammalian model (Tao et al., 2021), and has been experimentally validated for uncovering the virulence factors of *A. baumannii*. It is followed by *Caenorhabditis elegans (C. elegans)* (Mylonakis et al., 2016), zebrafish (*Danio rerio*) (Plumet et al., 2024), *Drosophila melanogaster (Drosophila)* (Qin et al., 2020), *Dictyostelium discoideum (D. discoideum)* (Iwashkiw et al., 2012), marine medaka (*Oryzias melastigma*) (Gong et al., 2022), *Zophobas morio* (Rakovitsky et al., 2024), and *Lucilia sericata* (McKenna et al., 2022) (**Figure 4**). Over the last decade (2015-2024), *G. mellonella* (354) and *C. elegans* (167) have been extensively utilized in *in vivo* studies of *A. baumannii* pathogenesis, whereas zebrafish were used intermittently (**Figure 4**).

In this review, we focus on the invertebrates *G. mellonella*, *C. elegans*, and zebrafish, as they have attracted significant interest from scientists for their use in discovery of virulence factors. These models offer distinct advantages over other *in vivo* non-mammalian animal models.

#### 4.2.1 *Galleria mellonella* model

**Figure 5:**
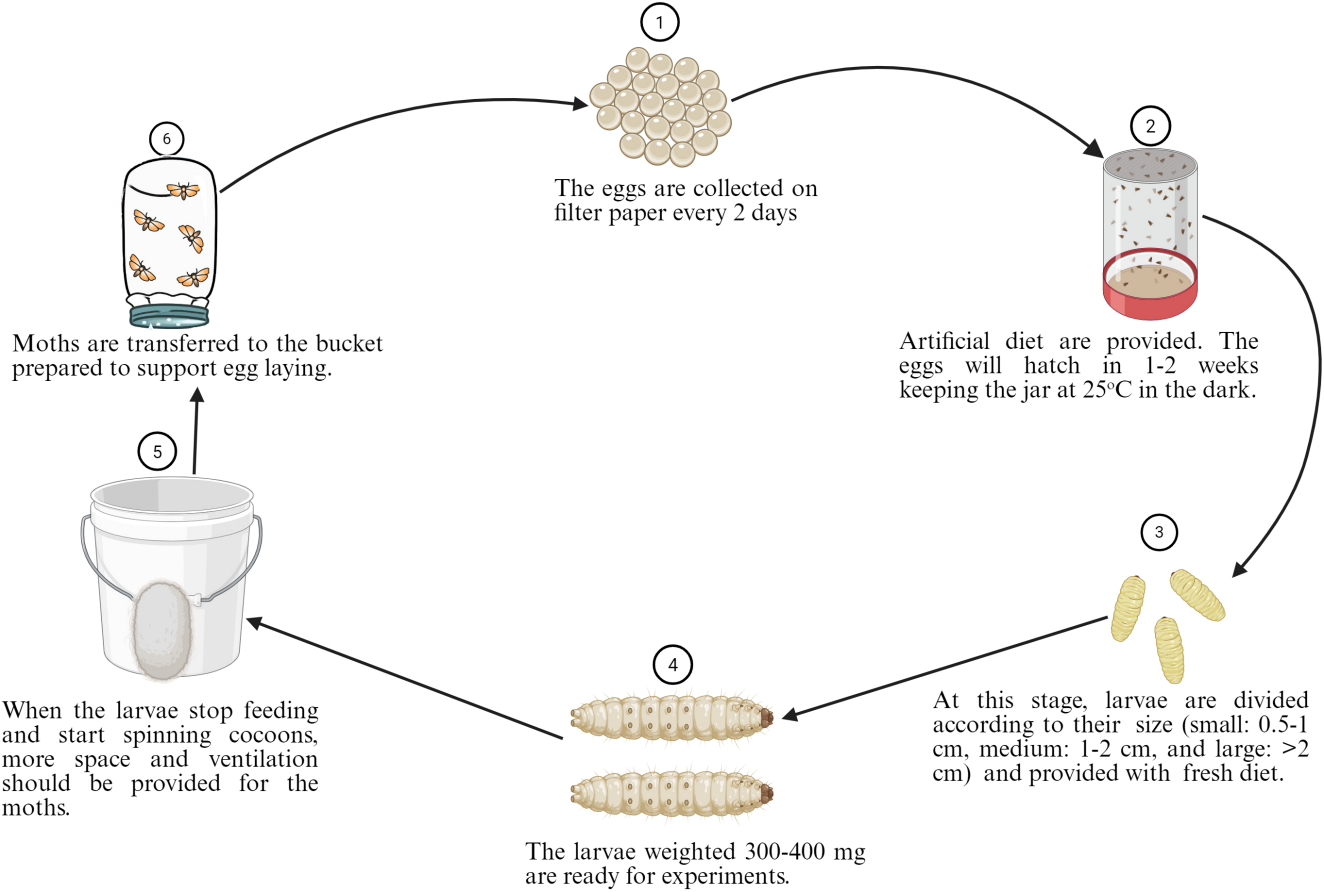
Steps for the standardized breeding of *G. mellonella* starting from eggs to moths. The composition of the optimized *G. mellonella* diet was composed of 54 g (9.5%) of rice flour, 54 g (9.5%) of oatmeal, 28 g (5%) of wheat germ, 84 g (14.8%) of torula yeast, 22 g (3.9%) of beeswax, 136 g (24%) of honey, 128 g (22.6%) of glycerol, and 60 g (10.7%) of water. The figure was created with BioRender (https://www.biorender.com/).

*G. mellonella*, the greater wax moth, has been utilized to study host-pathogen interactions in various organisms, including pathogenic bacteria and fungi. This larva is the most extensively used invertebrate model for investigating the host-pathogen interaction of *A. baumannii* (**Figure 4**), with 354 publications over the past decade. Most importantly, this larva offers many advantages over other non-mammalian models, as it mimics the human body. It can survive at 37 °C, which resembles human body temperature, a crucial factor for deciphering the virulence factors of human pathogens, such as *A. baumannii*. They possess innate cellular immunity (hemocytes) and humoral immunity (antimicrobial peptides, lysozymes, reactive oxygen species) but lack adaptive immune responses against bacterial infections (Kay et al., 2019). Furthermore, it can be purchased at a low cost, reproduces rapidly, and is easier to handle in the laboratory (**Figure 5**). The complete life cycle of the larva takes about 8–13 weeks. Mature, healthy larvae (2–3 cm in length) are used for experiments. *G. mellonella* does not require ethics approval for the studies. Considering this, *G. mellonella* has significant advantages in studying host-pathogen interactions and antibacterial efficacy. *G. mellonella* can be used for various purposes, including killing assays, bacterial burden assays, host immune response studies, and the isolation of bacterial or host RNA from infected larvae (Ten et al., 2023). **Figure 5** below illustrates the complete life cycle and mass production of *G. mellonella*.

Various arsenals of virulence factors of *A. baumannii* have been deciphered in this nonmammalian model. The T6SS is a critical key virulence factor in the host-*A. baumannii* interaction. The T6SS genes *tssB, tssD (hcp)*, and *tssM* were reported to be essential for *A. baumannii* pathogenesis, as their mutants impaired lethality in *G. mellonella* (Li P. et al., 2024). Additionally, the *TagP* (T6SS-Associated Gene PAAR) mutant strain was attenuated in numerous examined phenotypes, including its ability to cause disease in *G. mellonella*, showing a significant influence on pathogenicity (Li Y. et al., 2024). *Acinetobacter* trimeric autotransporter adhesin (*Ata*) is a key virulence factor of *A. baumannii*, exhibiting multiple functions including mediating adhesion and invasion. A *Δata* mutant strain was less effective in killing the larvae of *G. mellonella* (Weidensdorfer et al., 2019). Furthermore, disrupting the production of BasD and BauA, proteins vital for acinetobactin biosynthesis and transport, impaired the virulence of the ATCC 19606 strain. Infection assay of *G. mellonella* larvae demonstrated that impairing the acinetobactin-mediated iron acquisition system substantially reduced the killing ability of the ATCC 19606 strain (Gaddy et al., 2012).

The contribution of quorum-sensing (QS) genes to the pathogenesis of *A. baumannii* is also significant. The *abaM*, the third gene located between *abaI* and *abaR*, significantly influences the virulence of the *A. baumannii* 5075 strain, in which the killing ability of the *abaM::T26* mutant was diminished entirely in the killing assay of *G. mellonella* (López-Martín et al., 2021). Furthermore, scientists have reported that two-component system genes can regulate the pathogenesis of *A. baumannii*. For instance, the *adeAB* mutant of strain S1 substantially affected the virulence of this strain when *G. mellonella* was infected with 10^6^ CFU of bacteria (Richmond et al., 2016). The *SurA1*, the most dominant gene involved in biofilm formation, exhibited a decreased survival rate and limited dissemination when the *SurA1* mutant strain was injected into the *G. mellonella* infection model, indicating that this gene plays a significant role in the virulence of *A. baumannii* (Liu et al., 2016). The *gigAB* genes also play an essential role in the growth and virulence of this bacterium. The study revealed that when *G. mellonella* was inoculated with WT 17978, rapid larval killing commenced at 8 hpi. In contrast, no larval killing was observed within 48 hours of infection among those infected with the *ΔgigAB* mutant strain (Zhou et al., 2021), indicating that this gene is crucial in the pathogenesis of *A. baumannii*.

*G. mellonella* has also been utilized to study the virulence of *A. baumannii* by exposing the bacteria to various conditions, including antibiotics. For example, Schmitt et al. reported the virulence of persister and regular cells of *A. baumannii* after exposure to meropenem, and their study revealed that persister cells were more virulent than regular cells after injecting the cells into *G. mellonella* (Schmitt et al., 2023). Furthermore, the presence of *OXA-822* in *A. baumannii* 19606 significantly decreased the survival rate of infected larvae after treatment with meropenem compared to the control group without the *OXA-822* (Tietgen et al., 2021), indicating that meropenem is not protective in the presence of the *OXA-822* gene. The effect of bacterial biofilm formation on the survival of *G. mellonella* has also been reported. According to Khalil et al, the killing capacity of the strong biofilm-producing *A. baumannii* was considerably higher than that of the moderate and poor biofilm-producers (Khalil et al., 2021). Overall, *G. mellonella* has been extensively utilized in studying *A. baumannii* pathogenesis, and various virulent genes have been investigated using this model organism. Some of the recently examined virulent genes in *G. mellonella* are summarized in **Table 2**.

#### 4.2.2 Caenorhabditis elegans

The nematode *C. elegans* is one of the most utilized invertebrates for bacterial pathogenesis studies over the past decade. It has a short life cycle of about three days to grow and an overall lifespan of three weeks. *C. elegans* has been widely used in host-pathogen studies with several advantages, such as sharing extensive genome similarity (30%–60%) with mammals, including humans (Culetto and Sattelle, 2000); its small size; ease of cultivation and storage; being able to be humanized by transgenesis; lack of ethical concerns; and a transparent body, which allows real-time observation of what occurs inside the body of the nematode after bacterial infection, including *A. baumannii* (Vallejo et al., 2015). It has been used to study innate immune response to identify genes and signaling pathways, such as TIR-1, which is essential in activating the MAPK signaling pathway (Wang D. et al., 2024). Additionally, it has also been employed to assess the effectiveness of various antibacterial agents against both Gram-positive and Gram-negative bacteria, including multi-drug-resistant *A. baumannii*, their antibiofilm potencies, and toxic effects in nematode *C. elegans* (Chai et al., 2022; Khadke et al., 2021; Lee et al., 2019).

Most importantly, this nematode has been widely used to explore the virulence factors in host-pathogen interaction studies. According to Rumbo-Feal et al, inactivating the *A1S_0114* gene, which is involved in the production of acinetin 505, substantially reduced the virulence of the ATCC 17978 strain (Rumbo-Feal et al., 2017). The *abeD* mutant *A. baumannii* showed a significant reduction in virulence capability in *C. elegans* (Srinivasan et al., 2015), indicating that this gene contributes to the bacteria’s ability to withstand osmotic and oxidative stress and to kill the host. Reports also suggest that both RecA and CheW-like proteins are essential for full virulence of *A. baumannii*, as knocking out either gene impaired bacterial virulence in *C. elegans* (Corral et al., 2020).

The knockout of the Omp 33–36 kDa protein affected the virulence level of *A. baumannii* in the *C. elegans* infection model (Espinal et al., 2019). Likewise, deletion of the *blp1* gene impaired the virulence of *A. baumannii* during infection of the *C. elegans* (Skerniškytė et al., 2019a). This nematode is commonly used in killing assays to evaluate antimicrobial efficacy, analyze the virulence level of specific genes, and study host immune responses. It allows high-throughput screening of multiple bacterial strains more quickly and cost-effectively.

#### 4.2.3 Zebrafish (*Danio rerio*) model

Zebrafish is a small freshwater fish that has been primarily developed for embryogenesis studies. In the past decade, it has become a valuable model organism for studying human pathogens. This model offers several advantages, including small size, cost-effectiveness, ease of breeding, high reproduction, rapid development, and not being subject to ethical concern (Sullivan et al., 2017), in accordance with the 3Rs (Replacement, Reduction, and Refinement). Most importantly, transparency in their embryos facilitates visualization of host-microbe interactions and live imaging of infection dynamics (Schmitz et al., 2024). Indeed, this host model is excellent for immune response studies, because the zebrafish’s innate and adaptive immune systems shares considerable similarities with those of humans (H Meijer and P Spaink, 2011). During its first four days of life, exhibits only innate immunity. Its adaptive immune system becomes fully functional in 4–6 weeks (Lam et al., 2002). Thus, zebrafish is most suitable for studying innate immune responses in the first two weeks and adaptive immune responses later after about a month.

Zebrafish has been utilized to elucidate the role of the virulence factors of *A. baumannii*. For instance, the role of *AbaI* quorum sensing in pathogenesis has been examined in this model. Deletion of *AbaI* from the *A. baumannii* strain substantially decreased the virulence of the bacterium and recruitment of neutrophils to the infection site of zebrafish (Jiang et al., 2024). Compared to those infected with the wild-type strain, zebrafish infected with a strain lacking the carboxy-terminal processing protease (*Ctp*) gene showed a significantly higher survival rate (Roy et al., 2020), highlighting the importance of the *Ctp* gene in virulence. Additionally, zebrafish embryo has been used to demonstrate the virulence of eight capsule types. The strains with capsule types of K2, K9, K32, and K45 proved to be the most virulent; K1, K38, and K67 exhibited medium; and the K44 strain was less virulent (Neto et al., 2023). Another study demonstrated that *A. baumannii ΔgacS* and *ΔgacA* mutants had significantly reduced virulence in a zebrafish infection model compared to the wild-type strain (Bhuiyan et al., 2016), suggesting that these genes are crucial virulence regulators.

This host model has also been employed to investigate various therapeutic agents, including antimicrobials, antibiofilm formers, and anti-virulence, for their effective bactericidal activities, biofilm inhibition, and cytotoxicity level (Abirami et al., 2023; Jayathilaka et al., 2022).

Overall, due to their small size, ease of handling, cost-effectiveness, and lack of ethical concerns for non-mammalian animal models, zebrafish are highly suitable for experimental purposes. They address some challenges associated with *in vitro* cell culture. However, many gaps remain in the application of nonmammalian infection models for fully understanding of *A. baumannii* pathogenesis, such as growth at room temperature where bacterial pathogens may not express virulence factors, differences in target organs compared to humans, and the absence of complete mammalian immune responses. Therefore, confirming *in vivo* results from nonmammalian models with a mammalian model is essential to further understand bacterial pathogenesis and translating preclinical findings to clinical studies.

### 4.3 *In vivo* mammalian models

**Figure 6:**
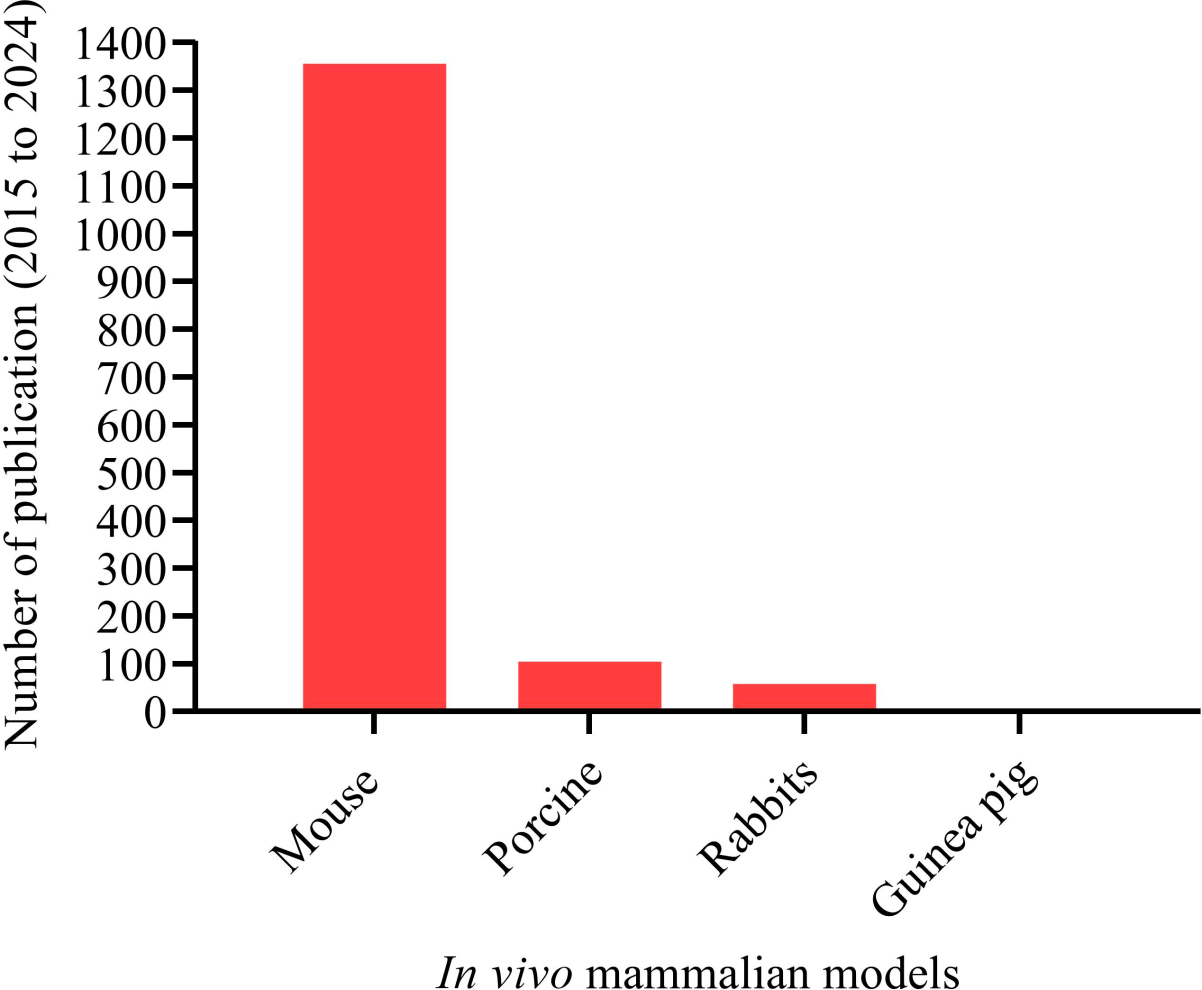
Mammalian animal models have been tested and established for studying *A. baumannii* pathogenesis. Total number of publications on mammalian animal host models and *A. baumannii* interaction. Mammalian animal models: “Mouse” (1,356); “Porcine” (105); “Rabbits” (58); and “Guinea pig” (3). A systematic search was made for the period from 2015 to 2024.

To address the challenges of nonmammalian models, mammalian models are crucial for a detailed understanding of the *A. baumannii* pathogenesis. Mammalian models offer significant advantages by closely mimicking human diseases, facilitating the investigation of potential virulence factors and the comprehensive testing of complex host-bacterial interactions. To date, mammalian animal models such as mice (Arrazuria et al., 2022), porcine (Zurawski et al., 2019), rabbits (Hansen et al., 2009), and guinea pigs (Bernabeu-Wittel et al., 2005) have been tested and established in studying *A. baumannii* pathogenesis (**Figure 6**). Over the last ten years (2015 to 2024), mice have been the paramount mammalian model extensively used in *in vivo* studies of *A. baumannii* pathogenesis, with 1,356 publications (**Figure 6**).

#### 4.3.1 Mouse model

We selected the mouse model to focus on in this review because scientists have consistently shown a strong interest in using mice as the primary and most common laboratory animal. Mice are used not only to decipher virulence factors but also for drug discovery, vaccine development, and anti-virulence approach studies. Over the last decade, more than 1,300 articles on *A. baumannii* pathogenesis using mouse models have been published. Porcine, rabbit, and guinea pig were less frequently used in *A. baumannii* studies compared to mice. However, these animals offer advantages, including anatomical and physiological similarities to humans. Their use in infectious disease research has decreased over the past decade due to certain limitations (**Figure 6**). Because of their large size, it isn’t easy to breed in large numbers, handle in a laboratory, and genetically modify (Mukherjee et al., 2022). Small animal models like mice are easier to breed in large quantities, handled in laboratory, and are suitable for genetic modification.

The use of mice as laboratory animals began with genetic research in the early 20th century and remains the main choice (**Figure 6**). Since humans and mice share over 90% of their genes, laboratory mice are essential in studying bacterial pathogenesis. They are crucial for identifying specific virulence factors associated with pathogens and understanding the immune responses elicited against them (Sarkar and Heise, 2019).

There are various types of mice used in biological research, including inbred, outbred, transgenic, knockout, and immunodeficient. Scientists have extensively used all except outbred mouse for studies of pathogen infection, including *A. baumannii* (Masopust et al., 2017). The inbred C57BL/6 and BALB/c are the most well-characterized and genetically stable mouse models for studying a variety of immunological and human pathogen studies, including *A. baumannii*.

Mouse models have been used for many years in studying specific virulence factors that contribute to the pathogenesis of microorganisms, including *A. baumannii* (Rumbo-Feal et al., 2017; Sams et al., 2012). In addition, they are essential to assess the effectiveness of new therapeutic agents and vaccines, where mouse models are immunized by the whole bacterial strain or specific bacterial cell components (such as virulence factors) and then assessed for any cytotoxicity and host immune response (Harris et al., 2013, 2019; Rafiei Delfan et al., 2024). Various types of mouse models can be utilized for diverse research objectives to mimic different types of infections. Common models include acute infection (intranasal, intraperitoneal, or intravenous routes) (Harris et al., 2013, 2019), pneumonia infection (Arrazuria et al., 2022; Ranjbar et al., 2022), wound infection (Ruamsap et al., 2022), sepsis model (Mirali et al., 2023), and immunocompromised mouse model (Huo et al., 2022; Kim et al., 2021), which are among the most commonly used mouse models in *A. baumannii* virulence studies.

Mouse models have been employed to identify virulence factors associated with the pathogenesis of *A. baumannii*. Outer membrane proteins are the most abundant surface proteins and key virulence factors in host-pathogen interactions, as explored in mouse models. For instance, the virulence of *OmpW, Omp34*, and *BauA* was elucidated and reported as potential vaccine candidates after confirming their effects using immunized mice (Gil-Marqués et al., 2022; Mirali et al., 2023). The synergistic effect of Omp34 and BauA was observed, with the combination providing greater protection than either protein alone in the immunized mice (Mirali et al., 2023). A substantial reduction of *A. baumannii* load was detected in the lungs, livers, and spleens vaccinated with Omp34 and BauA, indicating the potential virulence of these proteins (Mirali et al., 2023).

Investigation of other virulence factors using a mouse sepsis model revealed that virulence genes such as *bauE, abaI*, and *pgAB* played essential roles in the virulence of polymyxin B-resistant *A. baumannii*, indicating promising therapeutic targets (de Souza et al., 2024). Furthermore, pili are among the most important virulence factors for establishing *A. baumannii* infections. The Csu pili was known to mediate biofilm formation but was recently reported to enhance colonization of *A. baumannii* into various organs of mouse model such as liver, spleen, and lungs, which suggests it as a major virulence factor mediating *A. baumannii* adherence to initiate a disease in host models (Ahmad et al., 2023). The role of thioredoxin-A protein (*TrxA*) in *A*. *baumannii* biofilm formation has also been explored in the pulmonary infection model, and the mutant *TrxA* (Δ*TrxA*) was less virulent than the WT strain (May et al., 2019). Moreover, the transcriptomic analysis of the mutant revealed that loss of *feoA, mtnN, yfgC, basB, hisF, oatA*, and *bfnL* resulted in a significant loss of virulence in the mouse pneumonia model, highlighting these genes as promising therapeutic targets (Martinez-Guitian et al., 2021). The mice infected with a lethal dose of mutant strains showed higher survival rates compared to those infected with the WT strain. The virulence factors examined in mouse model in the last decade are summarized in **Table 3**.

## 5 Challenges and key considerations for model selection

Heterogeneity of strains is the most common challenge encountered in studying host and *A. baumannii* interactions. Due to its high genome plasticity, pan-genome analysis of 2,500 strains revealed nearly 20,000 different genes present across various *A. baumannii* strains (Mangas et al., 2019). It is important to note that different strains of *A. baumannii* employ distinct strategies to adhere, invade, internalize, and replicate within host cells. Some strains may possess specific virulence factors involved in pathogenesis or may share these factors with other strains. A comparative genomic analysis of 14,159 clinical isolates showed that only nine ST1791°/ST2^P^ strains carried 11 unique genes involved in pathogenesis, which are not found in other clinical strains (He et al., 2024). In contrast, Ata adhesin is present in 78% of all sequenced *A. baumannii* isolates, indicating that this gene is available in most sequenced strains (Weidensdorfer et al., 2019). The virulence factor *lasB* genes were present in 53.33% (16/30) of the strains, while *plcN* genes were present in 23.3% (7/30) of the strains. These differences suggest that the pathogenesis of the standard strains, ATCC 19606 and ATCC 17978, which are primarily used in numerous studies, may not accurately represent the recently circulating strains, as the genetic variability of most *A. baumannii* strains is significant.

Experimental variations in laboratory testing present another challenge in studying interactions between the host and *A. baumannii*. To explore how *A. baumannii* interacts with both animal and cell culture models, scientists have employed various animal models with different immune systems, varying bacterial doses, and different incubation periods after infection (**Tables 2** and **3**), which may complicate efforts to translate preclinical studies to clinical trials. Indeed, the virulence levels of the bacterial strains injected into the host models in these studies vary. When the bacterium is highly virulent, it can trigger a cytokine storm that even leads to the host’s death (Wang H. et al., 2024), highlighting the importance of managing host immune response to save the infected individuals. The infectivity level of *A. baumannii* influences the multiplicity of infection in cell culture models (**Table 1**). Different incubation durations and infection doses have been established and utilized for studying bacterial adhesion, invasion, and cell cytotoxicity.

When *G. mellonella* is used for survival studies or killing assays, the outcomes depend on several factors, including the number of *A. baumannii* cells inoculated into the larvae, incubation temperature, incubation time after injection, and the volume of injection. For instance, about 75% of the *G. mellonella* larvae inoculated with 3.7 × 10^5^ CFU of *A. baumannii* were killed after 48 hours, while very few were killed when inoculated with 3.7 × 10^4^ CFU, indicating that the killing ability depends on the inoculation dose (Peleg et al., 2009). This study also reported that the killing capacity of pathogenic *A. baumannii* is temperature-dependent. The rate of *G. mellonella* killing by *A. baumannii* was higher at 37 °C than at 30 °C (Peleg et al., 2009). Different studies used different incubation times (**Table 2**) and various injection volumes (Gaddy et al., 2012; Lin et al., 2023; Tang et al., 2020; Trebosc et al., 2022), which suggests that there are no standardized laboratory methods established for *A. baumannii* pathogenesis studies.

In laboratory experiments using a mouse infection model, various inoculum sizes per mouse have been employed so far (Gebhardt et al., 2020; Kim et al., 2021; Labrador-Herrera et al., 2020; Mirali et al., 2023). Highly diverse evaluation days after bacterial inoculation into mice have also been used to study the pathogenesis of *A. baumannii* in the mouse model (Ahmad et al., 2023; Belisario et al., 2021; Labrador-Herrera et al., 2020). There is also heterogeneity in immune responses against different *A. baumannii* strains with varying levels of virulence among both mammalian and nonmammalian models. Overall, no standardized methods, such as incubation durations and infection doses, are available for both cell culture and animal models. These heterogeneities could be among the major factors that hinder the translation from preclinical to clinical studies.

## 6 Conclusions

In this review, we focus on successful and well-established host models, including animal and cell culture models, to decipher the virulence factors of *A. baumannii*. Although many virulence factors have been identified and assessed for their abilities to stimulate immune responses using either *in vivo* animal models or *in vitro* culture systems, there is a gap in selecting the most appropriate models to elucidate the role of these factors in *A. baumannii* pathogenesis. This review compiles evidence that numerous cell culture and animal models have been thoroughly developed for studying the pathogenesis of *A. baumannii*. Over the past decade, A549, followed by HEK-293, HeLa, and HepG2 cells, have been the most successful, well-characterized, and extensively used cell lines in *A. baumannii* pathogenesis research. For studies on the inflammatory pathway, choosing immune-derived cells is essential. The RAW 264.7 macrophage and THP-1 monocytic cells are well-established immune cell models for studying inflammatory responses to *A. baumannii* infection. Among the nonmammalian models, *G. mellonella* larvae stand out as a more suitable model than others due to their capacity to be maintained at 37 °C, which is critical for host-pathogen interaction studies. *C. elegans* and zebrafish are also well-established nonmammalian models. Mice, as well-established mammalian models, provide diverse infection models that enable researchers to address varied objectives in *A. baumannii* pathogenesis studies. More works are needed to standardize methods, such as the selection of bacterial strains, initial bacterial inoculum, incubation duration, and follow-up time, when using both animal and cell culture models, as well as the multiplicity of infection in the case of cell culture systems. Notably, the 3D culture models, including organoid models, have yet to be developed for studying *A. baumannii* pathogenesis. Because 3D organoid models better replicate the physiology of human organs, they are crucial for understanding the complex interactions between host and pathogen. Overcoming these challenges could help translate preclinical findings into clinical applications.
